# Measuring antigen-specific responses in *Mycobacterium bovis*-infected warthogs (*Phacochoerus africanus*) using the intradermal tuberculin test

**DOI:** 10.1186/s12917-018-1685-8

**Published:** 2018-11-20

**Authors:** Eduard O. Roos, Francisco Olea-Popelka, Peter Buss, Guy A. Hausler, Robin Warren, Paul D. van Helden, Sven D. C. Parsons, Lin-Mari de Klerk-Lorist, Michele A. Miller

**Affiliations:** 1 NRF-DST Centre of Excellence for Biomedical Tuberculosis Research, South African Medical Research Council Centre for Tuberculosis Research, Division of Molecular Biology and Human Genetics, Faculty of Medicine and Health Sciences, Stellenbosch University, PO Box 241, Cape Town, 8000 South Africa; 2 Department of Clinical Sciences, College of Veterinary Medicine and Biomedical Sciences, Colorado State University, 300 W. Drake Rd, Fort Collins, CO 80523 USA; 3 Veterinary Wildlife Services, South African National Parks, Kruger National Park, Private Bag X402, Skukuza, 1350 South Africa; 4 Office of the State Veterinarian, Kruger National Park, Department of Agriculture, Forestries and Fisheries, PO Box 12, Skukuza, 1350 South Africa

**Keywords:** Bovine tuberculosis, Screening test, *Mycobacterium bovis*, Intradermal tuberculin test, Warthog, Wildlife

## Abstract

**Background:**

Bovine tuberculosis (bTB) caused by *Mycobacterium bovis* has previously been diagnosed in warthogs and infection can be highly prevalent (> 30%) in endemic areas. Thus, warthogs could potentially be an important species to consider as sentinels for disease surveillance. However, disease surveillance is dependent on availability of accurate diagnostic assays and only a few diagnostic tests have been investigated for warthogs. Furthermore, the tests that have been used in this species require laboratory equipment and trained personnel to obtain results. Therefore, this study investigated the use of the intradermal tuberculin test (ITT) to screen warthogs for bTB, which can be done with minimal equipment and under field conditions by most veterinarians and other qualified professionals. Changes in skin fold thickness measurements at the bovine purified protein derivative (PPD) administration site, between 0 and 72 h, were compared with differential changes between the bovine and avian PPD sites, for 34 warthogs, to evaluate the performance when different interpretation criteria for the ITT was used.

**Results:**

Using an increase of 1.8 mm or more at the bovine PPD site as a cut-off for positive responders, 69% of 16 *M. bovis* culture-positive warthogs had a positive test result, with 100% of the 18 culture-negative warthogs considered as test negative. When a differential of 1.2 mm or more in skin fold thickness at the bovine PPD compared to the avian PPD site was used as a cut-off for the comparative ITT, 81% of culture-positive warthogs were considered as test positive, with 100% of culture-negative warthogs considered as test negative.

**Conclusion:**

The findings in this study suggest that the ITT is a promising tool to use when screening warthogs for *M. bovis* infection.

**Electronic supplementary material:**

The online version of this article (10.1186/s12917-018-1685-8) contains supplementary material, which is available to authorized users.

## Background

The primary cause of bovine tuberculosis (bTB) is an acid-fast bacterium, *Mycobacterium bovis*, which has been reported to infect more than 17 wildlife species in South Africa [1]. The disease has become endemic in some nature reserves and private game farms within South Africa [2]. Furthermore, cases of bTB have been reported in threatened or endangered species, such as lions (*Panthera leo*) and rhinoceros (*Ceratotherium simum* and *Diceros bicornis*) [3, 4]. Certain wildlife species have become maintenance hosts of the disease in South Africa including the African buffalo (*Syncerus caffer*) [5]. Warthogs are also known to become infected with *M. bovis* and could potentially act as a maintenance host in endemic areas [6, 7]. This species is capable of crossing fences and other man-made barriers, which could lead to dissemination of disease, as is the case for wild boar (*Sus scrofa*), a bTB maintenance host in the Iberian Mediterranean ecosystem [1, 8]. Moreover, similar to feral pigs, warthogs may serve as a good sentinel as they are highly susceptible to this infection [9].

Accurate diagnostic tests are needed for disease surveillance. However, only a limited number of assays are available for bTB diagnosis in African wildlife species. A lack of approved laboratory facilities and logistical difficulties in getting samples to laboratories from disease controlled and remote areas also limits wildlife testing. One available method for field detection of bTB is to euthanase animals, identify granulomatous lesions on necropsy, and confirm the diagnosis using mycobacterial culture. However, it can take 6–8 weeks before culture results become available. Therefore, there is a need to have an accurate field-friendly ante-mortem assay for bTB screening of species such as warthogs, which can be readily performed by veterinarians.

The Intradermal Tuberculin Test (ITT) has been used for bTB detection in a range of species including domestic cattle, wild boar, white-tailed deer (*Odocoileus virginianus*), elk (*Cervus canadensis*), African buffalo, and lions [10–15]. The ITT is readily available to veterinarians and can be performed in the field, providing a result within 72 h. The objective of this study was to investigate the utility and test performance of the ITT for detection of *M. bovis* infection in warthogs.

## Results

**Fig. 1:**
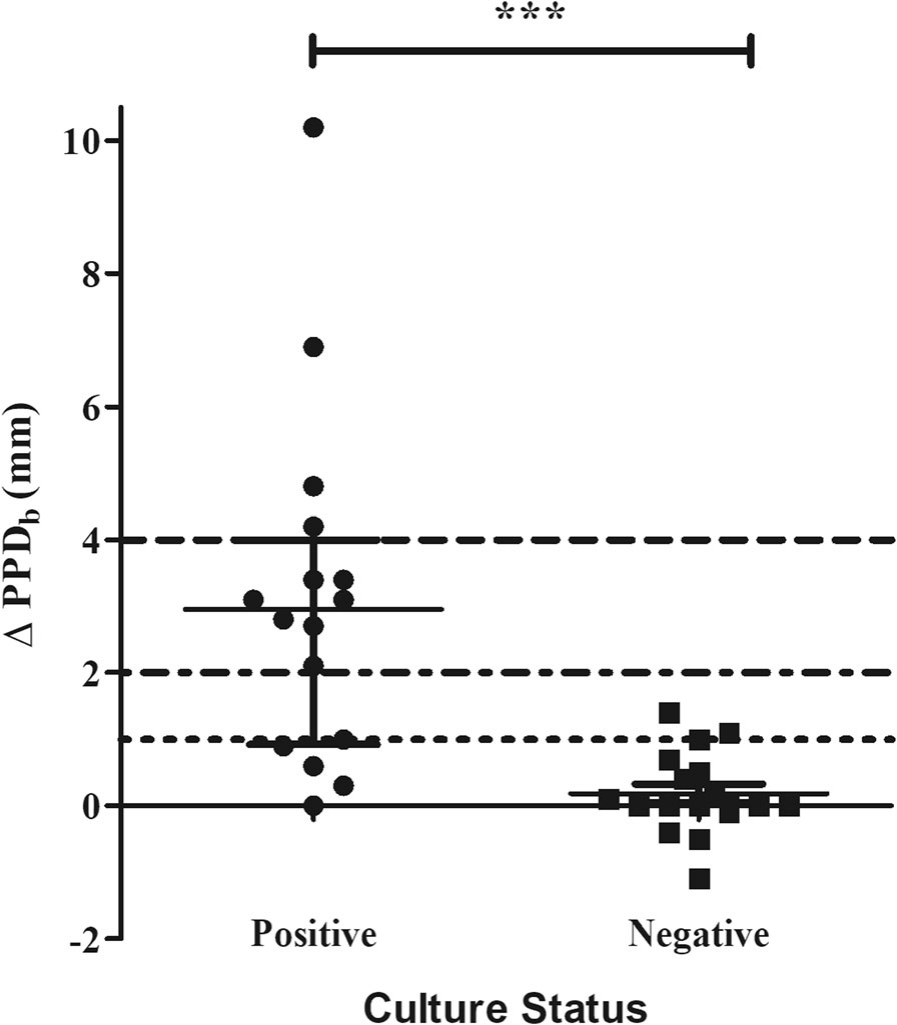
Differential in skin fold thickness (Δ PPD_b_) at the PPD_b_ injection site after 72 h in *M. bovis* culture-positive or culture-negative warthogs. Median and interquartile ranges are represented by the horizontal bars. The dotted lines represent the various published cut-off values used in other species (1, 2 and 4 mm). ** indicates *p* < 0.01, *** indicates *p* < 0.001

**Fig. 2:**
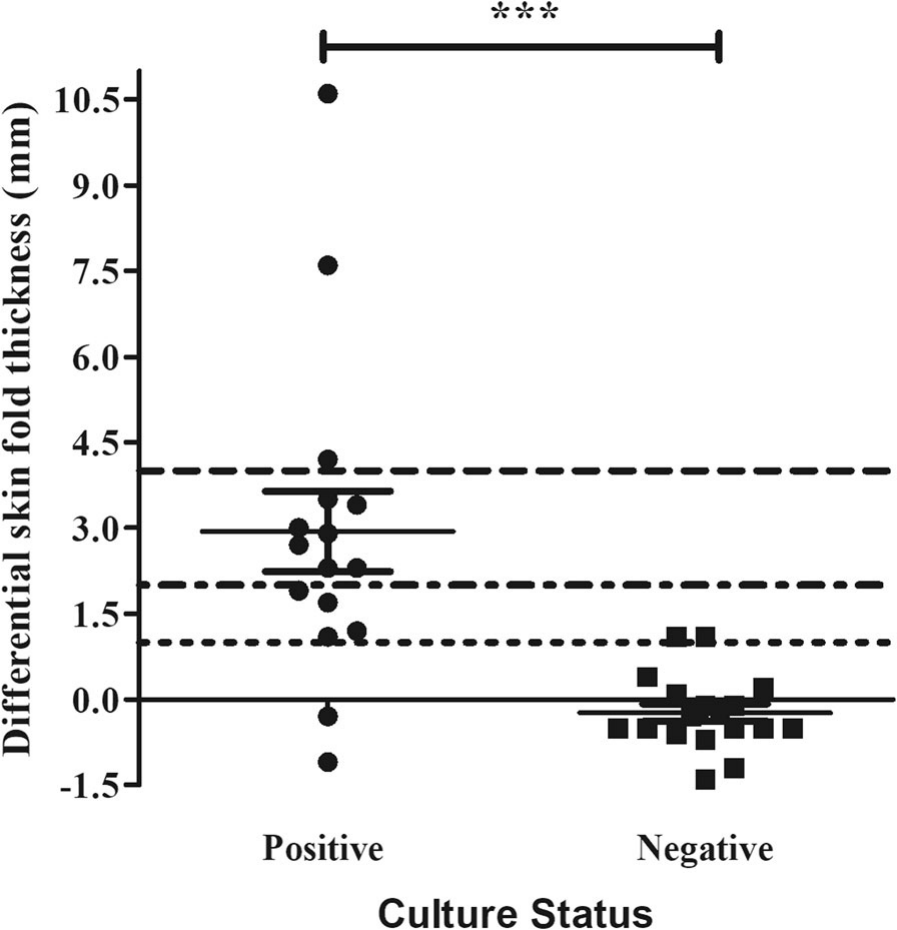
Test value of the skin test for *M. bovis* infected and uninfected warthogs. Test value was calculated by subtracting the PPD_a_ injection sites’ skin fold thickness from that of the PPD_b_ injection site after 72 h (PPD_b_ - PPD_a_). Median and interquartile ranges are represented by the horizontal bars. The dotted lines represent the various published cut-off values in other species (1, 2 and 4 mm). *** indicates *p* < 0.001

The ITT was measured for all 34 warthogs and individuals divided into two study cohorts based on mycobacterial culture results (Table 1). *M. bovis*-infection was confirmed by mycobacterial culture in 16 of the 34 warthogs. The SFT measurements from warthogs infected with NTMs were not significantly different from that of *M. bovis* culture-negative warthogs, for both PPD sites, and therefore grouped as culture-negative (*p* = 0.086). There was no significant difference in Δ PPD_a_ measurements between *M. bovis* culture-positive and culture-negative warthogs (*p* = 0.650, Additional file 1: Figure S1, Table 2). However, Δ PPD_b_ measurements were significantly greater for culture-positive compared to culture-negative animals (Fig. 1, Table 2). Furthermore, in *M. bovis* culture-positive warthogs, the increase in SFT at the PPD_b_ site (Δ PPD_b_) was significantly greater than at the PPD_a_ site (Δ PPD_a_) (*p* = 0.002), although no differences between these measurements were seen in culture-negative warthogs (*p* = 0.128, Additional file 1: Figure S1, Table 2). The PPD_b-a_ values were significantly greater for culture-positive warthogs compared to culture-negative warthogs (*p* < 0.0001, Fig. 2, Table 2).

**Table 1:** Raw data from 34 warthogs’ skinfold measurements (in mm) to PPD_b_ and PPD_a_ at time points 0 h and 72 h post-injection, the delta PPD_b_ and PPD_a_ as well as each warthogs’ mycobacterial culture result

Lab no.	PPD_b_ 0 h	PPD_b_ 72 h	Δ PPD_b_	PPD_a_ 0 h	PPD_a_ 72 h	Δ PPD_a_	Bacterial Culture
15/137	3	3	0	3.1	3.1	0	Culture negative
15/138	3.6	4.1	0.5	3.6	4.8	1.2	Culture negative
15/262	4.2	3.8	−0.4	4.6	5	0.4	Culture negative
15/271	4.9	4.9	0	4.3	4.8	0.5	Culture negative
15/302	2.6	4	1.4	2.7	4.6	1.9	Culture negative
15/304	3	3	0	3	3.3	0.3	Culture negative
15/307	5	6.1	1.1	4	6.6	2.6	Culture negative
15/308	3.3	3.3	0	3.7	3.8	0.1	Culture negative
15/310	3	3.2	0.2	3	3.4	0.4	Culture negative
15/305	3.2	4.2	1	2.8	3.1	0.3	*M. asiaticum*
15/516	5.3	4.2	-1.1	5.5	4.7	−0.8	*M. asiaticum*
15/309	3.2	3.6	0.4	3.3	3.4	0.1	*M. avium*
15/301	2.3	2.4	0.1	2.6	2.5	−0.1	*M. intracellulare*
15/515	3.9	3.4	−0.5	3.3	3	−0.3	*M. intracellulare*
15/535	3.8	3.8	0	5.5	5.2	−0.3	*M. paraffinicum*
15/514	4.8	4.7	−0.1	4	5.2	1.2	*M. simiae*
15/534	4.6	5.3	0.7	4.8	4.2	−0.6	*M. simiae*
15/536	4.5	4.5	0	4.5	5	0.5	*M. simiae*
15/140	4.8	6.9	2.1	4.6	4.6	0	*M. bovis*
15/248	3.8	8	4.2	4	3.8	−0.2	*M. bovis*
15/249	4.4	4.4	0	4.5	4.7	0.2	*M. bovis*
15/250	3.6	7	3.4	4.3	4.1	−0.2	*M. bovis*
15/251	4.8	5.7	0.9	4.1	4	−0.1	*M. bovis*
15/263	4.1	7.2	3.1	3.9	3.8	−0.1	*M. bovis*
15/264	3.8	14	10.2	3.2	3.4	0.2	*M. bovis*
15/265	4.3	4.9	0.6	3.8	3.8	0	*M. bovis*
15/266	4.3	11.2	6.9	4.2	3.6	-0.6	*M. bovis*
15/267	3.8	6.5	2.7	4.4	4.6	0.2	*M. bovis*
15/268	3.6	6.4	2.8	3.6	4.1	0.5	*M. bovis*
15/269	3.7	4.7	1	2.9	3.5	0.6	*M. bovis*
15/270	2.8	5.9	3.1	2.9	3.2	0.3	*M. bovis*
15/300	3.6	7	3.4	3.6	4	0.4	*M. bovis*
15/306	3.3	8.1	4.8	3.8	4.6	0.8	*M. bovis*
15/513	4.8	5.1	0.3	4.6	6.2	1.6	*M. bovis*

**Table 2:** Median values of the skinfold increase (in mm) using different combinations of measurements for the ITT in *M. bovis* culture-positive and culture–negative warthogs

Skinfold reading	Culture Positive	Culture Negative
Δ PPD_a_	0.2 mm (−0.1–0.5)	0.3 mm (−0.2–0.7)
Δ PPD_b_	3.0 mm (0.9–4.0)	0.0 mm (−0.1–0.6)
PPD_b_ - PPD_a_	2.5 mm (1.3–3.5)	-0.4 mm (− 0.5–0.1)

Interquartile ranges are shown in parenthese*s*

A warthog-specific cut-off value for the SITT was calculated as ≥1.8 mm using a ROC curve analysis (AUC = 0.91, 95% CI 0.81–1.0) (Additional file 2: Table S1). Based on this cut-off, 11 out of the 16 *M. bovis* culture-positive warthogs were SITT-positive (69%), while none of the 18 culture-negative warthogs had a positive test result (100%).

The cut-off value for the CITT was ≥1.2 mm (AUC = 0.91, 95% CI 0.79–1.0) (Additional file 3: Table S2). This cut-off value resulted in 13 of 16 culture-positive warthogs being CITT-positive (81%) and classified all 18 culture-negative warthogs as test negative (100%). No signs of oedema, heat, exudation or necrosis were observed at the PPD injection sites in any of the 34 warthogs tested.

## Discussion

This study shows that the ITT could distinguish between *M. bovis* culture-positive and negative warthogs from bTB endemic regions of South Africa, using both the SITT and CITT interpretations. Warthog specific cut-off values for the SITT and CITT were calculated to be ≥1.8 mm and ≥ 1.2 mm, respectively. The optimal ITT criterion for detection of infected warthogs in this study was the ≥1.2 mm cut-off for the CITT, which resulted in correct classification of 81% of culture-positive animals as CITT-positive and 100% of culture-negative warthogs as test negative. These results suggest that the interpretation of the ITT in this species should include the reaction to avian PPD (i.e., CITT) to identify the highest number of infected animals.

Importantly, diagnostic application in each species requires optimization and standardization of the ITT, as the injection site and dose may influence the delayed-type hypersensitivity response, with variable interpretation affecting test sensitivity [11, 12, 14, 16]. In this study, a double dose of tuberculin (0.2 ml PPD) was injected intradermally, caudal to each ear in warthogs, to minimise the chance of delayed-type hypersensitivity response failure due to dose, as is the case in lions and domestic cats [11].

The choice of ITT (SITT or CITT) is dependent on the prevalence and exposure of *M. bovis* as well as the presence of sensitising NTMs in a population [10]. The SITT is a simpler test, since it consists of one injection and measurement, although it lacks the discriminatory power of the CITT, where the response to PPD_a_ identifies sensitisation to NTMs [10]. In this study, the response at the PPD_a_ site was significantly less than that at the PPD_b_ site, indicating that the increase in SFT at the PPD_b_ site was a true measure of *M. bovis* infection and not a cross-reactive response to NTMs.

In many countries the initial recommendations that were in place for the ITT suggested the use of the single intradermal cervical test as the primary bTB screening test and the CITT as an ancillary test [10]. However, most of these countries have amended their regulations, as the CITT has been shown to be a more specific test than the SITT [17]. Furthermore, our results showed that the CITT was more sensitive than the SITT (81% versus 69%, correctly identifying animals with a culture-positive result). This may be due to the more sensitive cut-off value of ≥1.2 mm for the CITT compared to the ≥1.8 mm cut-off for the SITT [10, 18, 19]. It is important to note that neither the CITT nor SITT had any false positives in this study using these criteria.

Unfortunately, no biological test is perfect (i.e. has 100% sensitivity and specificity) and the CITT could not correctly classify all *M. bovis* culture-positive warthogs as test positive and at 81%, it is comparable to the sensitivity of the CITT in cattle [20]. The sub-optimal sensitivity could be due to various factors, for example, anergy, co-infection with NTMs, immunosuppression associated with nutritional, immobilization or transport stress, operator error, faulty equipment, or tuberculin not correctly administered intradermally [10].

For disease surveillance, the cut-off value of a screening test should be set to optimize sensitivity and specificity, after considering the prevalence of disease and epidemiological factors as well as clinical and financial constraints [19, 21]. A cost-effective and logistically feasible method is required for disease surveillance in wildlife since access to laboratories may be limited. Although previous reports have shown that serological assays can be used to identify infected warthogs [6], these require laboratory equipment and techniques which may not be readily available. Therefore, the ITT may be an alternative screening test to laboratory-based assays in some situations.

One limitation of this study was that cut-off values and test specificity were determined using endemic controls rather than animals from a known *M. bovis* negative population. Therefore, future research should ideally include an unexposed population of warthogs to evaluate specificity and determine a diagnostic cut-off value for the ITT.

## Conclusion

This study demonstrates that an antigen-specific in vivo response to *M. bovis* can be measured in warthogs when challenged with PPD_b_, thus confirming the usefulness of the ITT for this species. Cut-off values determined by ROC curve analyses were able to distinguish between *M. bovis* culture-positive and culture-negative warthogs with good sensitivity and specificity. Interpretation of the ITT under the criteria followed for the CITT, allowed greater numbers of infected warthogs to be detected. Thus, the application of the ITT will be a valuable tool for disease surveillance in warthogs.

## Methods

### Animals and sampling

In 2015, warthogs were captured and culled as part of drought management in the Greater Kruger National Park area (GKNP) by park veterinarians [22]. Since bTB is endemic in the GKNP, all warthogs in this study were considered exposed to *M. bovis*. Sixteen female and eighteen male warthogs were first immobilized and held in quarantine bomas to evaluate performance of the ITT in this species, as described below. Based on physical examination at the time of capture and post-mortem evaluation, all the animals were deemed to be healthy and in good to moderate body condition. The authors did not find evidence of any condition that would result in an immunocompromised animal, when the ITT was read. After reading the ITT, immobilized warthogs were humanely euthanized using succinylcholine (25 mg/kg; Kyron Laboratories, Benrose, South Africa), saturated with potassium chloride, administered intravenous. This drug was chosen since the warthog was already unconscious, the drug was readily available, could be administered in sufficient quantities to result in rapid death to a large animal, and did not present a health hazard if meat was consumed (by scavengers or others). Since the warthogs were part of a disease surveillance program, all animals were euthanized and a full necropsy performed. Post-mortem examination and tissue sampling were performed as previously described [6]. In summary, lymph nodes were collected from all warthogs and examined for gross lesions consistent with bTB. If no visible legions were observed, samples were pooled according to anatomical site and all sets cultured.

### Immobilization

All warthogs were immobilized using a drug combination of (i) zolazepam-tiletamine (Zoletil®; Virbac RSA, (Pty) Ltd., Centurion, South Africa) in combination with azaperone (Kyron Laboratories (Pty) Limited, Benrose, South Africa) or medetomidine (Kyron), or (ii) azaperone, butorphanol (Kyron), medetomidine and ketamine (Kyron) [22–24]. Immobilizations and holding conditions complied with the South African National Parks Standard Operating Procedures for the Capture, Transportation and Maintenance in Holding Facilities of Wildlife.

### Intradermal tuberculin test (ITT)

The intradermal tuberculin test was performed as described elsewhere [11]. Briefly, the skin fold thickness (SFT) caudal to each ear was measured using a spring loaded Hauptner calliper with pistol grip prior to administration of the purified protein derivative (PPD; Institute for Animal Sciences, Lelystadt, Netherlands) injection (Additional file 4: Figure S2). PPD was injected intradermally at 0 h: 0.2 ml bovine PPD (30,000 IU/ml) (PPD_b_) on the left and 0.2 ml avian PPD (25,000 IU/ml) (PPD_a_) on the right. After 72 h, the SFT at each PPD injection site was measured and examined for signs of oedema, heat, exudation or necrosis [25]. The same experienced operator performed all measurements and PPD injections.

The ITT was interpreted in two ways. For the single intradermal tuberculin test (SITT), the SFT prior to the PPD_b_ injection (0 h) was subtracted from the measurement at the same site 72 h post-injection (Δ PPD_b_). For the comparative intradermal tuberculin test (CITT), the 72 h SFT at the PPD_a_ injection site was subtracted from that at the PPD_b_ injection site (PPD_b-a_). This was done since the degree of dehydration varies between individuals. Thus, only the absolute increase in response was used to calculate the PPD_b_ specific response. The degree of dehydration in individual animals has also been recognized by the South African Department of Agriculture, Forestry and Fisheries in TB testing of African buffaloes [26]. The Δ PPD_a_ was calculated in the same way as the Δ PPD_b_.

### Mycobacterial cultures and speciation

All tissue samples were processed using the BACTEC™ MGIT™ 960 system (BD Biosciences, New Jersey, USA) as previously described [13]. Positive cultures in the MGIT system were further analysed by Ziehl-Neelsen (ZN) staining and all ZN-positive cultures were speciated using genetic region of difference analysis [27] and 16S DNA sequencing [28]. The status of warthogs as *M. bovis*-infected or uninfected was based on culture results.

### Data analysis

Statistical analyses were performed using GraphPad Prism version 5 (GraphPad Software, March 2007). The Δ PPD_b_ and Δ PPD_a_ values for *M. bovis* culture-positive and culture-negative animals were compared within and between groups using a Kruskal-Wallis statistic with a Dunn’s Multiple Comparison test. The Δ PPD_a_ and Δ PPD_b_ comparisons were done to confirm the specific response of *M. bovis* culture-positive warthogs to the PPD_b_ injection. Evaluation of the Δ PPD_a_ was done to provide information as to whether non-tuberculous mycobacteria (NTM) influence the PPD_b_ responses. The SITT results for culture-positive and culture-negative animals were compared using a Mann Whitney test, as were results for the CITT. Warthog specific cut-off values to determine positive responders were calculated using a receiver operator characteristic (ROC) curve analysis and selected based on the Youden’s index [29].

## Additional files

Additional file 1: **Figure S1.** Differential in skin fold thickness at the PPD_b_ and PPD_a_ (Δ PPD_b_ and Δ PPD_a_) injection site after 72 h in *M. bovis* culture-positive (CP) or culture-negative (CN) warthogs. Median and interquartile ranges are represented by the horizontal bars. ** indicates *p* < 0.01, *** indicates *p* < 0.001. (TIF 614 kb)

Additional file 2: **Table S1.** Receiver operator characteristics curve analysis data for Δ PPD_b_. Warthog specific cut-off values and respective sensitivity and specificity, with 95% CI in parentheses. Youden’s index for each cut-off value is also indicated. (DOCX 15 kb)

Additional file 3: **Table S2.** Receiver operator characteristics curve analysis data for PPD_b_ – PPD_a_. Warthog specific cut-off values and respective sensitivity and specificity, with 95% CI in parentheses. Youden’s index for each cut-off value is also indicated. (DOCX 14 kb)

Additional file 4: **Figure S2.** Purified protein derivative injection site for the intradermal tuberculin test, caudal to each ear. The picture shows the appropriate use of the callipers to measure the skin fold thickness (mm). (PNG 1964 kb)
