# Supplementary figures and images for: Measuring antigen-specific responses in Mycobacterium bovis-infected warthogs (Phacochoerus africanus) using the intradermal tuberculin test
Source: BMC Vet Res. 2018 Nov 20;14:360. doi: 10.1186/s12917-018-1685-8 (PMC6247514; doi:10.1186/s12917-018-1685-8)

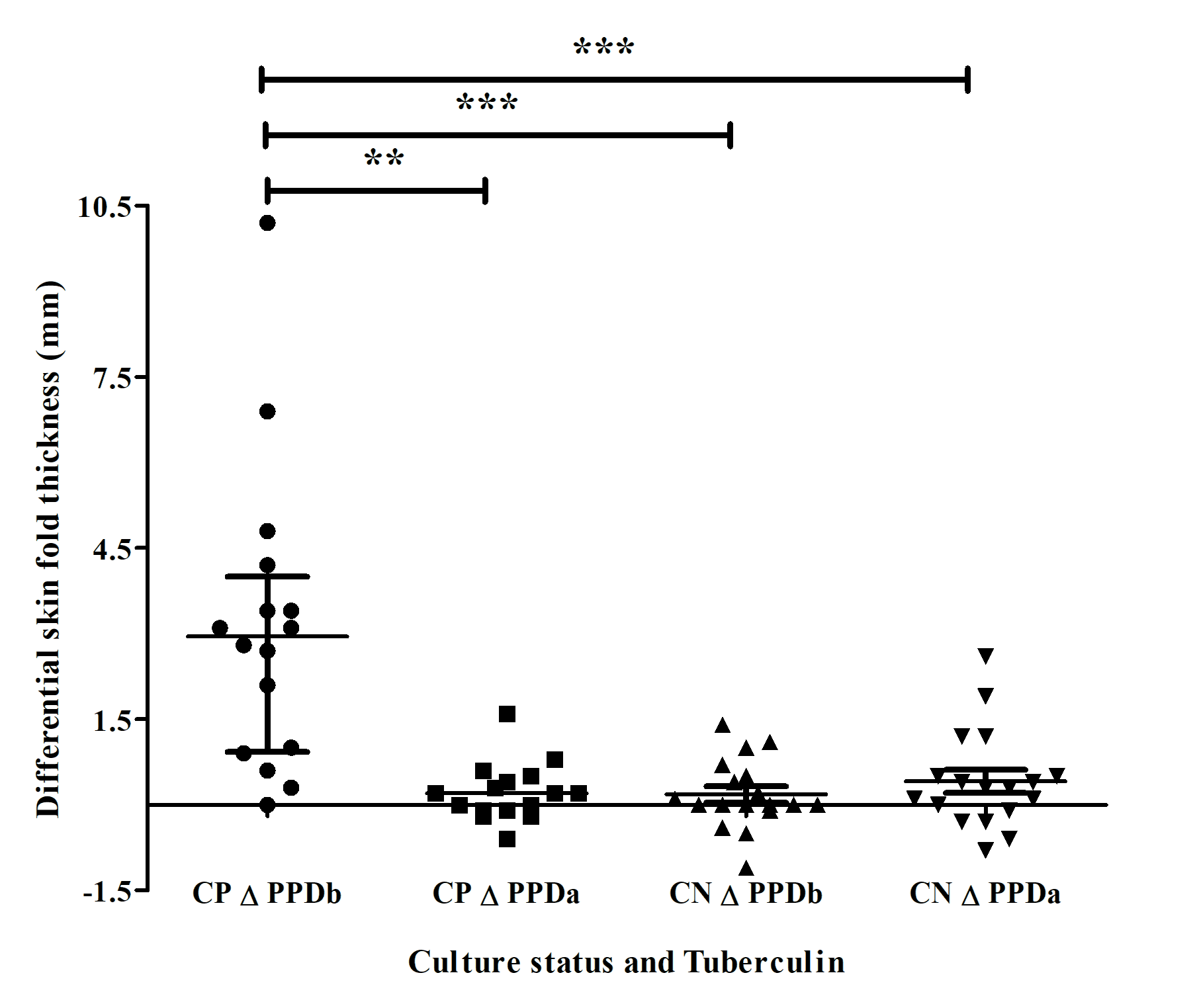

Supplement: Supplementary file 1 — Figure S1. Differential in skin fold thickness at the PPDb and PPDa (Δ PPDb and Δ PPDa) injection site after 72 h in M. bovis culture-positive (CP) or culture-negative (CN) warthogs. Median and interquartile ranges are represented by the horizontal bars. ** indicates p < 0.01, *** indicates p < 0.001. (TIF 614 kb) [file 12917_2018_1685_MOESM1_ESM.tif]

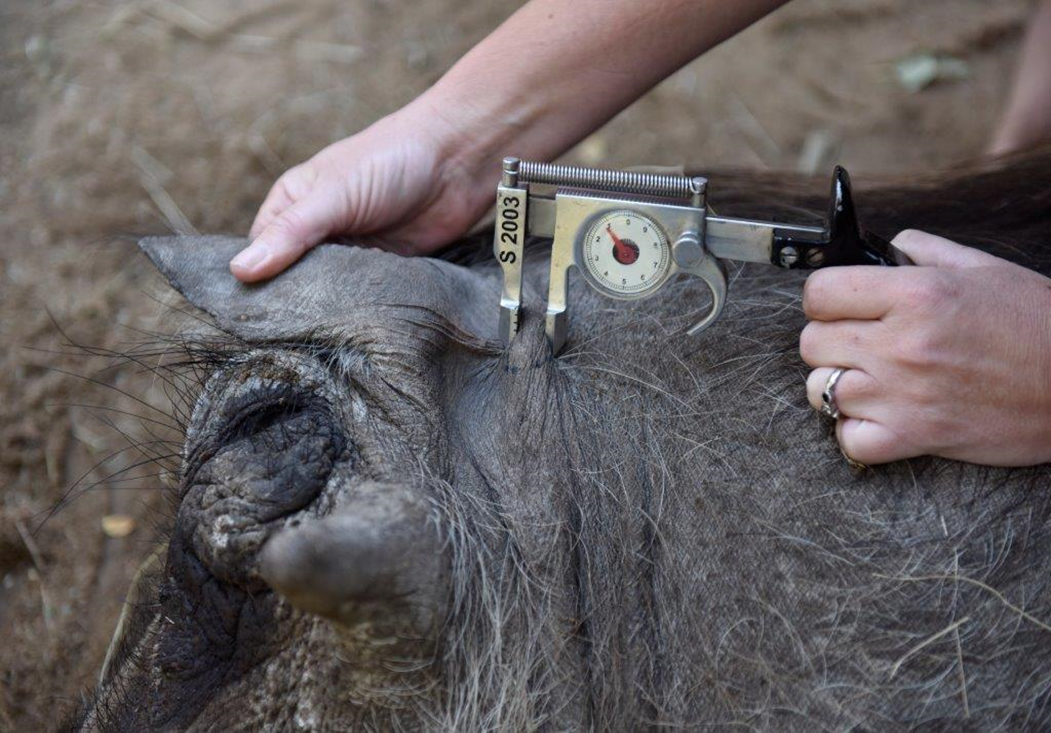

Supplement: Supplementary file 4 — Figure S2. Purified protein derivative injection site for the intradermal tuberculin test, caudal to each ear. The picture shows the appropriate use of the callipers to measure the skin fold thickness (mm). (PNG 1964 kb) [file 12917_2018_1685_MOESM4_ESM.png]
