# Supplementary material for: Measuring antigen-specific responses in Mycobacterium bovis-infected warthogs (Phacochoerus africanus) using the intradermal tuberculin test
Source: BMC Vet Res. 2018 Nov 20;14:360. doi: 10.1186/s12917-018-1685-8 (PMC6247514; doi:10.1186/s12917-018-1685-8)
Supplement: Supplementary file 2 — Table S1. Receiver operator characteristics curve analysis data for Δ PPDb. Warthog specific cut-off values and respective sensitivity and specificity, with 95% CI in parentheses. Youden’s index for each cut-off value is also indicated. (DOCX 15 kb) [file 12917_2018_1685_MOESM2_ESM.docx]

**Table S1** Receiver operator characteristics curve analysis data for Δ PPD_b_. Warthog specific cut-off values and respective sensitivity and specificity, with 95% CI in parentheses. Youden’s index for each cut-off value is also indicated.

| Cut-off | Sensitivity | Specificity | Youden's index |
| --- | --- | --- | --- |
| > 0.1 | 100 (79-100%) | 56 (31-79%) | 49 |
| > 0.2 | 94 (70-100%) | 61 (36-83%) | 55 |
| > 0.3 | 94 (70-100%) | 67 (41-87%) | 60 |
| > 0.4 | 88 (62-98%) | 68 (41-87%) | 54 |
| > 0.5 | 88 (62-98%) | 72 (47-90%) | 60 |
| > 0.6 | 88 (62-98%) | 78 (52-94%) | 65 |
| > 0.7 | 81 (54-96%) | 79 (52-94%) | 59 |
| > 0.8 | 81 (54-96%) | 83 (59-96%) | 65 |
| > 1.0 | 75 (48-93%) | 84 (59-96%) | 58 |
| > 1.1 | 69 (41-89%) | 89 (65-99%) | 58 |
| > 1.3 | 69 (41-89%) | 94 (73-100%) | 63 |
| > 1.8 | 69 (41-89%) | 100 (81-100%) | 69 |
| > 2.4 | 63 (35-85%) | 100 (81-100%) | 63 |
| > 2.8 | 56 (30-80%) | 100 (81-100%) | 56 |
| > 3.0 | 50 (25-75%) | 100 (81-100%) | 50 |
| > 3.3 | 38 (15-65%) | 100 (81-100%) | 38 |
| > 3.8 | 25 (7-52%) | 100 (81-100%) | 25 |
| > 4.5 | 19 (4-46%) | 100 (81-100%) | 19 |
| > 5.9 | 13 (2-38%) | 100 (81-100%) | 13 |
| > 8.6 | 6 (0-30%) | 100 (81-100%) | 6 |
