# Supplementary material for: Measuring antigen-specific responses in Mycobacterium bovis-infected warthogs (Phacochoerus africanus) using the intradermal tuberculin test
Source: BMC Vet Res. 2018 Nov 20;14:360. doi: 10.1186/s12917-018-1685-8 (PMC6247514; doi:10.1186/s12917-018-1685-8)
Supplement: Supplementary file 3 — Table S2. Receiver operator characteristics curve analysis data for PPDb – PPDa. Warthog specific cut-off values and respective sensitivity and specificity, with 95% CI in parentheses. Youden’s index for each cut-off value is also indicated. (DOCX 14 kb) [file 12917_2018_1685_MOESM3_ESM.docx]

**Table S2** Receiver operator characteristics curve analysis data for PPD_b ­_– PPD_a_. Warthog specific cut-off values and respective sensitivity and specificity, with 95% CI in parentheses. Youden’s index for each cut-off value is also indicated.

| Cut-off | Sensitivity | Specificity | Youden's index |
| --- | --- | --- | --- |
| > 0.2 | 88 (62-99%) | 78 (52-94%) | 65 |
| > 0.3 | 88 (62-99%) | 83 (59-96%) | 71 |
| > 0.8 | 88 (62-99%) | 89 (65-99%) | 76 |
| > 1.2 | 81 (54-96%) | 100 (81-100%) | 81 |
| > 1.5 | 75 (48-93%) | 100 (81-100%) | 75 |
| > 1.8 | 69 (41-89%) | 100 (81-100%) | 69 |
| > 2.1 | 63 (35-85%) | 100 (81-100%) | 63 |
| > 2.5 | 50 (25-75%) | 100 (81-100%) | 50 |
| > 2.8 | 44 (20-70%) | 100 (81-100%) | 44 |
| > 3.0 | 38 (15-65%) | 100 (81-100%) | 38 |
| > 3.2 | 31 (11-59%) | 100 (81-100%) | 31 |
| > 3.5 | 25 (7-52%) | 100 (81-100%) | 25 |
| > 3.9 | 19 (4-46%) | 100 (81-100%) | 19 |
| > 5.9 | 13 (2-38%) | 100 (81-100%) | 13 |
| > 9.1 | 6 (0-30%) | 100 (81-100%) | 6 |
